# Modified laparoscopic lateral suspension with a five-arm mesh in pelvic organ prolapse surgery

**DOI:** 10.1186/s12905-021-01388-0

**Published:** 2021-06-15

**Authors:** Eren Akbaba, Burak Sezgin

**Affiliations:** Obstetrics and Gynecology, Faculty of Medicine, Muğla Sıtkı Koçman University, Muğla, Turkey

**Keywords:** Pelvic organ prolapse (POP), Laparoscopic lateral suspension (LLS), Synthetic T-shaped mesh, Five-arm mesh, Posterior compartment repair

## Abstract

**Background:**

Laparoscopic lateral suspension (LLS) is a laparoscopic technique used to treat pelvic organ prolapse (POP) in apical and anterior compartment defect with the use of a synthetic T-shaped mesh graft. The posterior compartment is repaired using a second mesh or a procedure along with LLS, such as posterior colporrhaphy. The aim of this study was to evaluate the clinical results of LLS for POP using a five-arm mesh instead of a T-shaped mesh graft to repair the defect of the posterior compartment in addition to the apical and anterior compartments.

**Methods:**

Data from 37 patients with a diagnosis of advanced-stage (≥ 3) POP undergoing LLS with the use of a five-arm mesh were retrospectively analysed. Pre-operative and post-operative examinations and, surgical outcomes were determined. The results of measurements and examinations, reoperation rates, erosion rates, lower urinary tract symptoms, and complications were analysed. The Prolapse Quality of Life Questionnaire (P-QOL) was also used.

**Results:**

The median post-operative follow-up was 20 (13–34) months. There was a significant improvement in POP-Q scores in all treated compartments, with overall objective cure rates of 94.5% for the apical compartment, 86.4% for the anterior compartment, and 91.8% for the posterior compartment. The median operative time was 96 (76–112) minutes. The median length of hospitalization was 2 (1–3) days. A significant improvement in vaginal bulge, urinary urgency, incomplete voiding, urinary frequency, and constipation was observed after surgery. The sexuality among patients increased from 13 (35.1%) preoperatively to 22 (59.4%) post-operatively. De novo stress urinary incontinence developed in 7 (18.9%) patients. The P-QOL scores improved significantly after surgery.

**Conclusions:**

In advanced-stage POP patients, the posterior compartment damage can also be repaired in LLS with the use of a single five-arm mesh without the need for an additional procedure, and the recurrence rate can be reduced.

## Background

Pelvic organ prolapse (POP) is a downward protrusion of one or more uterine or vaginal parts (anterior or posterior vaginal wall, uterus [cervix], or apex of the vagina [vaginal vault or cuff scar after hysterectomy]) [1]. The prevalence of POP is 3–6% or 41–50% in postmenopausal women when defined and graded based on symptoms or examination respectively [2]. The lifetime risk of surgery for women with POP is 12–19%, and 10–30% of those women require reoperation [3].

Various vaginal and abdominal surgical approaches using native tissue or mesh have been used for the treatment of POP. Following vaginal mesh withdrawal announcements of the U.S. Food and Drug Administration in 2009 and 2011 regarding POP repair, trans‐abdominal mesh procedures have become more popular [4, 5]. Sacrocolpopexy (SCP) is the first and most preferred laparoscopic technique for treating POP. However, its operative time is long, and its learning curve is steep. These techniques require dissection at the level of the promontory or sacral area, which can be challenging, particularly in obese women. Sacral area injuries can lead to serious neurological, ureteral, or vascular injuries [6, 7].

The laparoscopic lateral suspension (LLS) procedure described by Dubuisson et al. does not require dissection at the level of the promontory or sacral area. Therefore, the risk of severe complications is lower. LLS uses a synthetic T-shaped mesh graft and can be performed with or without hysterectomy or in hysterectomized women [8]. Data on LLS in the anterior and apical compartments suggest an objective success rate of > 90% after 1 year [9, 10]. LLS can be an alternative to SCP for the repair of the apical compartment [11, 12]. The posterior compartment is repaired using a second mesh or a procedure a long with LLS, such as posterior colporrhaphy [13]. To contribute to the determination of the best method for the surgical treatment of POP, we modified LLS using a five-arm mesh instead of a synthetic T-shaped mesh graft to repair the defect of the posterior compartment together with the apical and anterior compartments in non-hysterectomized patients with advanced-stage (≥ 3). In the current study, we presented the modified LLS and aimed to analyse the clinical results of this modified procedure.

## Methods

### Ethical approval

This retrospective study was conducted in the Department of Obstetrics and Gynecology of the Faculty of Medicine of Muğla Sıtkı Koçman University, Muğla, Turkey. Ethical approval was obtained from the Faculty’s Ethics Committee (no. 2/II; 20 January 2021). The study was conducted according to the recommendations of Helsinki declaration. Written informed consent was obtained from all patients before undergoing surgery.

### Study design

Between March 2016 and January 2020, we performed LLS using a five-arm mesh in 49 patients diagnosed with advanced-stage (≥ 3) POP. Patients undergoing previous POP surgery (4) with a vaginal mesh, lack of medical records (8) were excluded. A total of 37 patients who had stage ≥ 3 uterovaginal prolapse were included in the study.

Demographic and clinical characteristics were retrieved from electronic medical records, imaging results, and preoperative, intraoperative, and post-operative notes. The data included the simplified pelvic organ prolapse quantification (POP-Q) stage and degree of prolapse (points Ba, Bp, and C) [1] and prolapse-related symptoms.

### Surgical technique

**Fig. 1:**
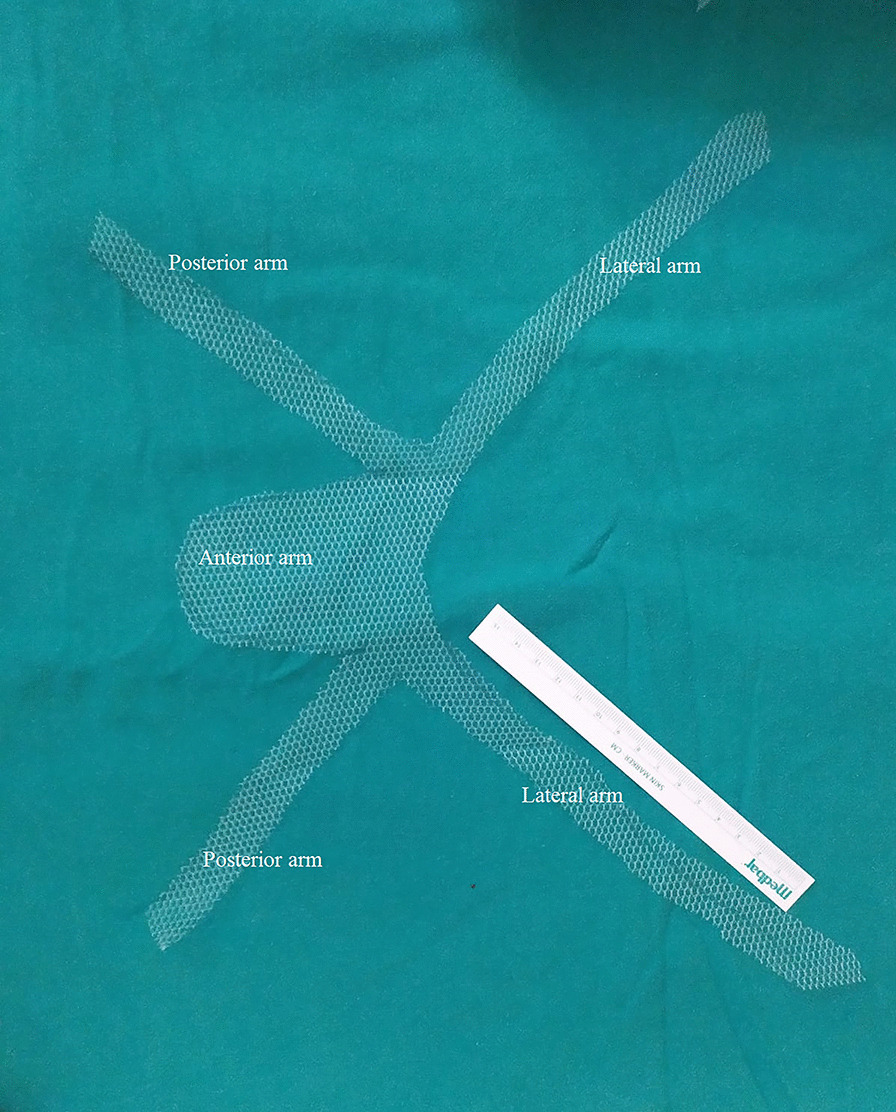
Appearance of 5 arm mesh

All operations were performed by a single surgeon (EA). Surgery was performed under general anaesthesia in the Trendelenburg position. A polypropylene macropore mesh (Parietene™, Sofradim-Covidien, Trévoux, France) 30 × 30 cm in size was cut with scissors, and a five-arm mesh with an anterior rectangular part approximately 4 × 6 cm in size, two long arms 2 × 18 cm in size, and two short arms 2 × 6 cm in size was prepared (Fig. 1). We used a central 10-mm umbilical trocar for the zero-degree optic and three 5-mm trocars (lower right, lower left, and upper left quadrants of abdomen). A RUMI®II retractor (CooperSurgical, Trumbull, CT, USA) was inserted into the uterine cavity for uterine manipulation. The vesicovaginal space was dissected until border of the lower third of the vagina by directing the uterus, cervix, and partially the vagina with the retractor. The rectovaginal space was dissected. Then, bilateral windows with a diameter of 1.5 cm were opened in the avascular area of the ligamentum latum leaves.

**Fig. 2:**
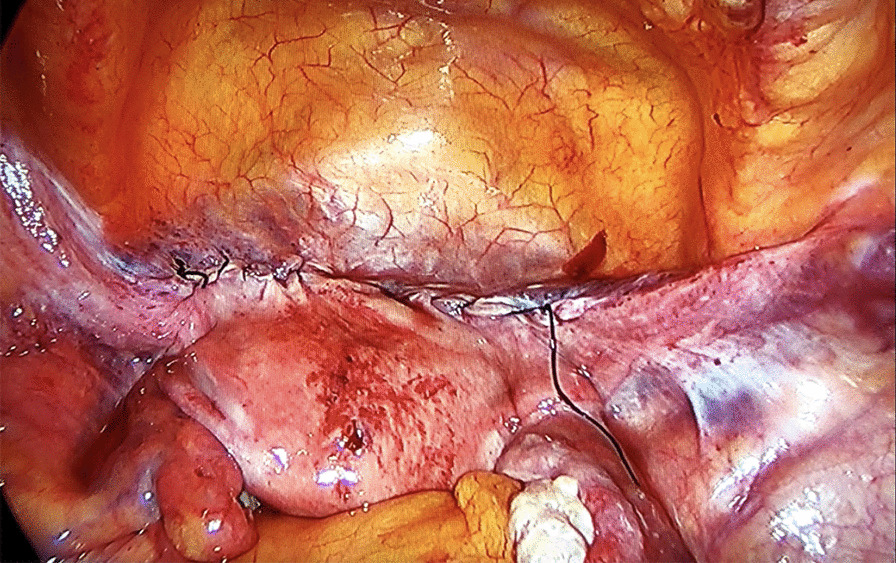
Anterior appearance after peritonization

**Fig. 3:**
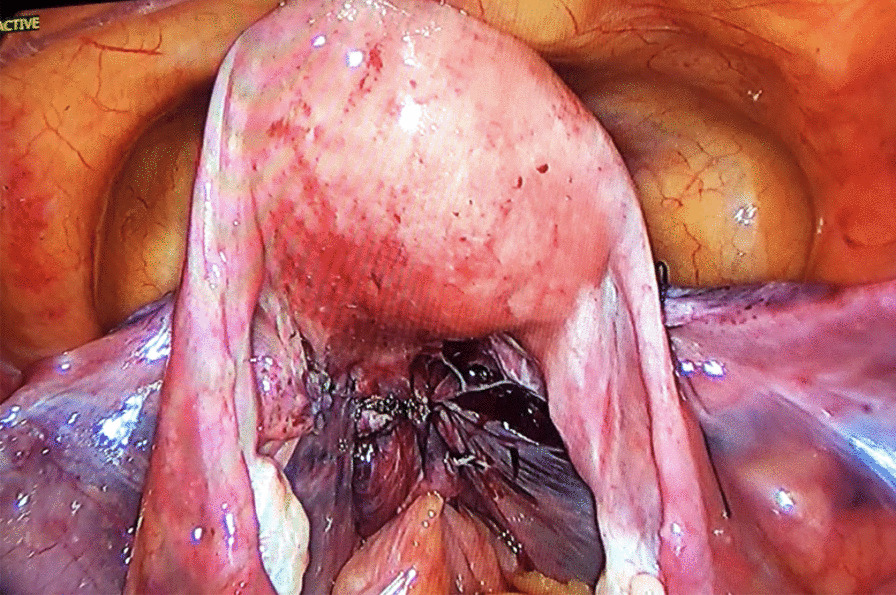
Posterior appearance after peritonization

The anterior part of the mesh was placed in the vesicovaginal space and sutured separately to the anterior vaginal wall and the cervical and isthmus parts of the uterus with no. 2–0 Prolene® (monofilament polypropylene suture; Ethicon, Somerville, NJ, USA) to prevent shrinkage of the mesh. At this stage, an absorbable tucker fixation device (AbsorbaTack™ [ABSTACK30X]; Covidien) was also used to fix the mesh. Two lateral arms of the mesh were passed through the windows opened on the ligamentum latum leaves and behind the uterus bilaterally and sutured separately to the rectovaginal fascia, sacrouterine ligament, and posterior vaginal wall with a no. 2–0 Prolene suture. A 3-mm skin incision was performed on both sides 2 cm above the iliac crest and 4 cm posterior to the anterior superior iliac spine. A laparoscopic grasper was advanced into the avascular area by inspecting the large vessels (external iliac artery and veins) in the retroperitoneal area and passing under the ligamentum rotundum. Then, the tip of one of the long arms (2 × 18 cm) of the mesh was pulled out of the skin. The same procedure was repeated on the other side. A symmetrical lateral suspension was performed. Following the “tension-free” repair principle, the lateral arms of the mesh were not sutured to the fascia. The mesh was then cut at the level of the skin before the closure of the incision. The parts of the mesh that were placed in the vesicovaginal and rectovaginal spaces were closed by peritonization using a no. 0 absorbable Vicryl Rapide™ (polyglactin 910; Ethicon) suture (Figs. 2 and 3).

### Post-operative analysis

Urogynaecological examinations were performed in the lithotomy position and additionally by performing the Valsalva manoeuvre in the standing position. The examinations included grading and POP-Q staging. The surgical outcomes were determined according to the recommendations of the International Urogynecological Association [14]. The results of measurements and examinations, reoperation rates, erosion rates, lower urinary tract symptoms (LUTS), and complications were recorded.

Satisfactory anatomic objective cure was defined as a POP-Q score of ≤ − 1. Complications were evaluated according to the Clavien–Dindo classification and classified according to the joint International Urogynecological Association/International Continence Society (IUGA/ICS) complication classification [15, 16]. A validated Turkish version of the Prolapse Quality of Life Questionnaire (P-QOL) was used to assess the patients’ quality of life [17]. This questionnaire included questions for general health perceptions, prolapse impact, role limitations, physical/social limitations, personal relationships, emotions, sleep/energy and severity measures, A lower score represents a better quality of life (range 0–100).

### Statistical analysis

The data were analysed using IBM SPSS Statistics version 20.0 (IBM, Armonk, NY, USA) for Windows. The Shapiro–Wilk test was used to evaluate data normality. Continuous data were reported as means ± standard deviations, medians and ranges, and medians and 25th/75th percentiles. Categorical data were reported as numbers and percentages. For intergroup comparisons, Wilcoxon signed-rank test was used. The post hoc power analysis was 98%, indicating adequate power in our study to demonstrate a significant difference in our technique. A *p* value of < 0.05 was considered statistically significant.

## Results

The patients’ demographic data, preoperative examination findings, and prior POP-related surgery histories are summarized in Table 1.

**Table 1:** Preoperative demographic and clinical characteristics of the patients (n = 37)

Variable	Value
Age (years), mean ± SD	56.03 ± 9.93
BMI (kg/m^2^), mean ± SD	28.99 ± 3.03
Parity, median (range)	4 (1–9)
Number of vaginal deliveries, median (range)	3 (1–9)
Menopausal status, n (%)	
Premenopausal	8 (21.62)
Postmenopausal	29 (78.38)
Prior POP surgery, (n)	
Anterior colporrhaphy	4
Manchester–Fothergill	2
Prior stress urinary incontinence surgery, (n)	
Transobturator sub-urethral sling	2
Kelly–Kennedy	2
POP-Q stage, n (%)	
3	16 (43.24)
4	21 (56.76)

*SD* Standard deviation, *BMI* Body mass index, *POP* Pelvic organ prolapse, *POP-Q* Pelvic organ prolapse quantification

The pre and post-operative anatomic outcomes are summarized in Table 2. The median post-operative follow-up was 20 (13–34) months. There was a significant improvement in POP-Q scores in all treated compartments, with overall objective cure rates of 94.5% for the apical compartment, 86.4% for the anterior compartment, and 91.8% for the posterior compartment. The median operative time was 96 (76–112) minutes. The median length of hospitalization was 2 days.

**Table 2:** Anatomical outcomes

POP-Q point	Preoperative median (Q1, Q3)	Post-operative median (Q1, Q3)	*p*
Bp	4 (3, 4)	− 5 (− 6, − 3)	0.001*
C	5 (4, 6)	− 7 (− 7, − 6)	0.001*
Ba	3 (3, 4)	− 3 (− 3, − 2)	0.001*

*POP-Q* Pelvic Organ Prolapse Quantification, *Q1* First quartile (25th percentile), *Q3* Third quartile (75th percentile)

*Significant at the 5% level (Wilcoxon signed-rank test)

A comparison of pre- and post-operative symptomatic outcomes is shown in Table 3. The most common symptom was palpable swelling in the genital region and a consequent walking difficulty. A significant improvement in vaginal bulge, urinary urgency, incomplete voiding, and urinary frequency was observed after surgery. An improvement in constipation symptom was also noted. Furthermore, the number of sexually active patients increased from 13 preoperatively to 22 post-operatively. Three (13.6%) of these 22 patients had dyspareunia. Occult stress urinary incontinence (SUI) was noted in two patients preoperatively, and de novo SUI developed in seven patients post-operatively. The P-QOL scores improved significantly after surgery (Table 3).

**Table 3:** Comparison of pre- and post-operative symptomatic outcomes

Variable	Preoperative	Post-operative	*p*
Vaginal bulging, n (%)	37 (100)	2 (5.4)	0.001*
Urinary urgency, n (%)	24 (64.8)	3 (8.1)	0.001*
Incomplete voiding, n (%)	29 (78.3)	3 (8.1)	0.001*
Urinary frequency, n (%)	27 (72.9)	8 (21.6)	0.001*
Stress urinary incontinence, n (%)	2 (5.4)	7 (18.9)	0.025*
Constipation, n (%)	11 (29.7)	3 (8.1)	0.005*
Faecal incontinence, n (%)	4 (10.8)	2 (5.4)	0.157
Sexual activity, n (%)	13 (35.1)	22 (59.4)	0.007*
Dyspareunia, n (%)	6 (16.2)	3 (8.1)	0.180
Pelvic pain, n (%)	11 (29.7)	8 (21.6)	0.221
P-QOL score, median (Q1, Q3)	75 (50–75)	25 (0–50)	0.001*

*POP-QOL* Prolapse Quality of Life Questionnaire, *Q1* First quartile (25th percentile), *Q3* Third quartile (75th percentile)

*Significant at the 5% level (Wilcoxon signed-rank test)

The post-operative complications are shown in Table 4. No major complications were noted (Clavien-Dindo grade 1).

**Table 4:** Post-operative data and complications

Variable	Value
Post-operative follow-up (months), median (range)	20 (13–34)
Operative time (minutes), median (range)	96 (76–112)
Length of hospital stay (days), median (range)	2 (1–3)
Reoperations due to recurrence, n (%)	
Anterior compartment	2 (5.4)
Apical compartment	0 (0)
Posterior compartment	0 (0)
Vaginal mesh erosion, n (%)	1 (2.7)
Reoperation for SUI, n (%)	4 (10.8)

*SUI* Stress urinary incontinence

Cystocele occurred in three patients (Ba + 1, + 2 and + 2), and rectocele developed in two patients (Bp + 1, + 3). Anterior colporrhaphy was performed in the two patients with cystocele recurrence, as they were symptomatic. The patient with rectocele (stage ≥ 3) recurrence did not undergo surgery due to being asymptomatic. Anterior vaginal wall mesh exposure grade 2 (> 1 cm) was observed in one patient. The part of the vaginal wall with mesh exposure was determined at the fifth postoperative month and classified as 3BT3S according to the IUGA/ICS Prosthesis/Graft Complication Classification System. The exposed part was resected, and the vaginal mucosa was primarily repaired. Four of the seven patients with de novo SUI underwent retropubic tension-free vaginal tape procedures, whereas the other three opted for conservative treatment methods.

## Discussion

POP is accompanied by anatomic symptoms such as palpable swelling and bruising in the genital region and causes dysfunctions such as incontinence and difficulty in defaecation and micturition, as well as sexual dysfunctions. Due to their nature, these dysfunctions and symptoms exert serious negative effects on patients’ psychological well-being and social life [18]. Restoring compartment defects in POP close to normal anatomy can contribute to mitigating these effects [19].

A study reported 1-year anatomic success rates of 88.2% for the anterior, 86.1% for the apical, and 80.8% for the posterior compartment after LLS [20]. In this study, we used a five-arm mesh in 37 LLS procedures in stage ≥ 3 POP patients. The analysis of POP-Q stages showed a statistically significant improvement of anatomic defects. The best outcome was noted in the apical compartment, with a 94.5% success rate. The success rate in the anterior compartment was 86.4%. All patients had posterior compartment defects preoperatively, which improved at a rate of 91.8% postoperatively. While the apical and anterior compartment outcomes achieved with a five-arm mesh were similar to those achieved with SCP and LLS, the posterior vaginal repair outcomes of five-arm mesh were better than those achieved with LLS [7, 9].

LLS is not indicated in the case of significant concomitant apical and posterior defects (such as enterocele or high rectocele) [21]. A previous study reported a reduced risk of reoperation in patients undergoing apical compartment defect repair when anterior and posterior compartment repair was simultaneous performed [22]. The lateral arms of the synthetic T-shaped mesh graft used in LLS do not ensure the closure of the pouch of Douglas. This may lead to the progression of the posterior defect [9].

To repair apical compartment defects together with posterior compartment defects or to prevent de novo posterior defects, posterior colporrhaphy with native tissue is performed, or a posterior compartment repair procedure using a mesh is added to SCP or LLS [11]. In hysterectomized POP patients undergoing SCP or LLS with a four-arm mesh placed in the apical compartment and sutured to the deep posterior vaginal wall into the rectovaginal space, an additional posterior repair procedure is not needed [23, 24].

The risk of mesh-related complications increases with the size of the mesh [25]. In their first attempt in non-hysterectomized patients, Dubuisson et al. performed LLS with two separate meshes 14 × 3 cm in size placed in the anterior and posterior compartments [8]. Later, Dubuisson et al. used a T-shaped mesh with a middle part 5–8 cm long and 4–6 cm wide and arms 3 cm wide to repair apical and anterior compartment defects and a rectangular polyester patch 6–8 cm long and 4–6 cm wide fixed to the rectovaginal fascia to repair posterior compartment defects but did not perform suspension [11]. We repaired the posterior compartment defect by suturing two short arms of a five-arm mesh 6 cm long and 2 cm wide to the sacrouterine ligament, the posterior wall of the cervix, and the posterior vaginal wall. When we elevated the long arms, a symmetrical suspension was achieved not only in the anterior and apical compartments but also in the posterior compartment. The Bp point was at a distance of − 5 cm during follow-ups of at least 1 year. Posterior compartment prolapse recurred only in three (8.1%) patients. Dubuisson et al. performed apical and anterior compartment repairs in 73 POP patients. They cut from a 25 × 25 cm polypropylene mesh and obtained two long arms 15–20 mm wide and a rectangular piece 4–7 cm wide. They used a mesh of the same size in the posterior compartment simultaneously. Their analysis showed a post-operative posterior wall recurrence rate of 11% [26].

A meta-analysis found that SCP with hysterectomy is associated with a fourfold increase in the risk of mesh exposure compared to SCP without hysterectomy [27]. In another study, the success rate was lower and the recurrence and mesh erosion rates were higher in POP patients undergoing hysterectomy along with LLS than in patients not undergoing hysterectomy [28]. We did not perform any hysterectomies with a prolapse indication. A five-arm mesh can easily be used in LLS without the need for hysterectomy.

In this study, we observed an improvement in symptoms along with anatomic improvements. A marked improvement in vaginal bulge, urinary urgency, incomplete voiding, urinary frequency, constipation, and faecal incontinence was noted. Previous studies have reported de novo constipation rates of 1.9–11.4% in patients undergoing abdominal SCP and 5.5–8.4% in patients undergoing LLS [12, 29]. In our study, post-operative constipation was observed in 8.1% of the patients. The incidence of SUI is estimated to be 20% in patients with a POP diagnosis and higher in advanced-stage POP patients [30]. Veit-Rubin et al. reported a rate of 5.2% for SUI after LLS in patients with POP grade 2–4 [12]. In our study, the rate of de novo SUI was 18.9%. The reason why our rate for SUI is higher than the literature may be due to the fact that all of our cases were advanced stage POP (stage 3–4).

LLS seems to preserve or restore normal sexual function. Not performing simultaneous hysterectomy is associated with more favourable outcomes [20]. In our study, although the quality of sexual function was assessed based on patients’ self-reports, the number of sexually active patients appears to have increased, and the rate of dyspareunia appears to have decreased post-operatively.

Although the use of a mesh in POP surgery reduces recurrence rates, complications such as mesh-related vaginal erosion, granulomas, dyspareunia, vesicovaginal fistulas, and aggravation of bladder overactivity cannot be ignored. To reduce the risk of mesh erosion, it is important to select the appropriate mesh type (macroporous and monofilamentous polypropylene). Moreover, aggressive dissection, which may deteriorate perfusion, should be avoided, and care should be taken not to damage surrounding organs, such as the urinary bladder and rectum [31]. Vaginal erosion is the most common mesh-related complication [32]. Although it can be treated with conservative methods, it may also require complex and repeated surgical interventions [33]. The risk of mesh erosion is 5 times higher in patients undergoing POP or urinary incontinence surgery. A posterior mesh is associated with a higher risk of erosion than an anterior mesh [34]. A systematic review that included more than 7000 women undergoing abdominal POP surgery found a median mesh erosion rate of 4% during an average follow-up of 2 years [35]. In our study, mesh erosion occurred in the anterior compartment (1.5 cm) in one patient (2.7%).

The main limitations of our study were the retrospective design, relatively small sample size, and subjective assessment of sexual activity. The strengths of our study are that all operations were standardized and performed by a single surgeon. To the best of our knowledge, the current study is the first to investigate the efficacy of modified LLS with a five-arm mesh in pelvic organ prolapse surgery.

## Conclusion

In advanced-stage POP patients undergoing LLS with the use of a five-arm mesh, damaged compartments, including the posterior compartment, can be repaired without the need for an additional procedure, and the recurrence rate can be reduced. Randomized controlled trials are required to determine the best surgical method for POP treatment.

## Data Availability

The datasets used and/or analysed during the current study available from the corresponding author on reasonable request.

## Abbreviations

POP: pelvic organ prolapse
LLS: laparoscopic lateral suspension
SCP: sacrocolpopexy
LUTS: lower urinary tract symptoms
POP-Q: pelvic organ prolapse quantification
SUI: stress urinary incontinence
PQOL: Prolapse Quality of Life Questionnaire

## Declarations

### Competing of interests

E.Akbaba and B.Sezgin declare that they have no competing of interests**.**
